# Nutritional Importance of Carotenoids and Their Effect on Liver Health: A Review

**DOI:** 10.3390/antiox8070229

**Published:** 2019-07-19

**Authors:** Laura Inés Elvira-Torales, Javier García-Alonso, María Jesús Periago-Castón

**Affiliations:** 1 Department of Food Technology, Food Science and Nutrition, Faculty of Veterinary Sciences, Regional Campus of International Excellence “Campus Mare Nostrum”, Biomedical Research Institute of Murcia (IMIB-Arrixaca-UMU), University Clinical Hospital “Virgen de la Arrixaca”, University of Murcia, Espinardo, 30071 Murcia, Spain; 2 Department of Food Engineering, Tierra Blanca Superior Technological Institute, Tierra Blanca 95180, Mexico

**Keywords:** β-carotene, lycopene, lutein, β-cryptoxanthin, non-alcoholic fatty liver disease (NAFLD), hepatic steatosis

## Abstract

The consumption of carotenoids has beneficial effects on health, reducing the risk of certain forms of cancer, cardiovascular diseases, and macular degeneration, among others. The mechanism of action of carotenoids has not been clearly identified; however, it has been associated with the antioxidant capacity of carotenoids, which acts against reactive oxygen species and inactivating free radicals, although it has also been shown that carotenoids modulate gene expression. Dietary carotenoids are absorbed and accumulated in the liver and other organs, where they exert their beneficial effects. In recent years, it has been described that the intake of carotenoids can significantly reduce the risk of suffering from liver diseases, such as non-alcoholic fatty liver disease (NAFLD). This disease is characterized by an imbalance in lipid metabolism producing the accumulation of fat in the hepatocyte, leading to lipoperoxidation, followed by oxidative stress and inflammation. In the first phases, the main treatment of NAFLD is to change the lifestyle, including dietary habits. In this sense, carotenoids have been shown to have a hepatoprotective effect due to their ability to reduce oxidative stress and regulate the lipid metabolism of hepatocytes by modulating certain genes. The objective of this review was to provide a description of the effects of dietary carotenoids from fruits and vegetables on liver health.

## 1. Introduction

In recent decades, carotenoids (lycopene, β-carotene, lutein, zeaxanthin, and β-cryptoxanthin) have aroused great interest in the field of human nutrition, as they act as biological antioxidants, contributing to the defense of the organism against reactive oxygen species (ROS) [1,2] and play a protective role in conditions, such as diabetes and CVD [3], impacting cellular signaling pathways and influencing the expression of certain genes, and inhibiting specific enzymes involved in the development of certain forms of cancer [4]. Dietary carotenoids are mainly accumulated in the liver, where they are transferred to be transported by the different lipoproteins for their release into the blood circulation and thus to be deposited and stored in different organs and tissues, such as the kidneys, adipose tissue, adrenal glands, testes, skin, and the prostate [5]. Adipose tissue (abdominal fat) is an important reserve site for carotenoids, showing a strong association between the intake of carotenoids and the concentrations of these antioxidants in plasma [6]. The carotenoids present in the skin can protect against the damaging effects of radiation and neutralize the attacks of free radicals, particularly ROS [7,8]. In addition, the concentrations of these carotenoids in the skin can increase with their dietary supplementation and decrease in people with oxidative stress, such as smokers. Similarly, carotenoids in plasma and skin decrease with exposure to UV rays [9]. The accumulation of these antioxidants, as well as of their metabolites in the liver, can exert a positive effect on the hepatocyte metabolism, regulating the cellular oxidative state in certain liver pathologies. Non-alcoholic fatty liver disease (NAFLD) is currently considered one of the most frequent chronic liver diseases in the world and represents a serious and growing clinical problem in developed and developing countries [10,11]. NAFLD can occur in different states, from simple steatosis to non-alcoholic steatohepatitis (NASH) with liver fibrosis and cirrhosis, which can eventually lead to hepatocellular carcinoma [12,13,14]. Ingestion of carotenoid-type antioxidants through the diet is considered as one of the possible mechanisms in the treatment of non-alcoholic fatty liver disease (NAFLD), thus avoiding the progression of NASH and other types of liver diseases [13,15,16,17,18,19]. In this review, we focused on the preventive potential of carotenoids at a nutritional level and their effect on liver health.

## 2. Carotenoids 

Carotenoids are lipophilic pigments synthesized by plants, fungi, algae, and bacteria [20,21]. In plants, carotenoids contribute to the photosynthetic system and protect them against photodamage, in addition to helping in the production of phytohormones [22]. As pigments, they are responsible for the red, orange, pink, and yellow colors of the leaves of plants, fruits, vegetables, and some birds, insects, fish, and crustaceans [23,24,25,26]. More than 750 types of carotenoids have been identified in nature, but only about 100 are present in detectable amounts within the human diet [20]. Between 30 and 40 carotenoids have been found in human blood samples and the six most abundant carotenoids consist of more than 95% of the carotenoids found in blood plasma: lycopene, lutein, β-carotene, β-cryptoxanthin, α-carotene, and zeaxanthin [27,28].

### 2.1. Chemical Structure

Typically, carotenoids are composed of forty carbon atoms formed by the union of eight isoprene units covalently linked. These structures can be completely linear or have rings at one or both ends, and these rings can contain hydroxyl groups, ketones, epoxies, or others. Carotenoids belong to two structural groups: carotenes containing carbon and hydrogen atoms and xanthophylls containing at least one oxygen atom [29,30]. In addition, carotenoids can be classified into two categories: carotenoids with provitamin A activity (β-carotene and β-cryptoxanthin) and carotenoids without provitamin A activity (lycopene and lutein) [31]. The different carotenoids originate basically by modification in the base structure, cyclization of the final groups, and by the introduction of oxygen groups that give them their characteristic colors and antioxidant properties [23].

### 2.2. Source of Carotenoids

Fruits and vegetables are the main sources of carotenoids in the human diet, providing 80–90% of these compounds in developed countries and 82% in developing countries [32,33]. Since carotenoids cannot be synthesized in the human body, they are used as biomarkers to reflect the intake of fruits and vegetables, establishing a direct relationship between the consumption of vegetables and the concentration of carotenoids in blood [34,35].

Carotenoids are found in almost all foods of plant origin, but Britton and Khachik [36] established a classification of dietary sources according to their carotenoid content, establishing sources with a low content (0–0.1 mg/100 g fresh product), moderate (0.1–0.5 mg/100 g fresh product), high (0.5–2 mg/100 g fresh product), and very high content (>2 mg/100 g fresh product).

β-carotene is the main carotenoid present in the human diet. It is found mainly in yellow–orange and dark green fruits and vegetables, such as carrots, squash, spinach, papaya, mango, apricots, and sweet potatoes [37,38]. Lycopene is a carotenoid that lacks provitamin A activity and is responsible for red to pink colors in fruits and vegetables, such as tomatoes, red grapefruit, watermelon, apricots, pink guava, and papaya [12,39]. Tomatoes and tomato-based products are the most common sources of lycopene in the human diet and account for more than 85% of the dietary intake of this carotenoid in North America [13]. Likewise, in the European diet, the intake of tomato-based lycopene and tomato products (canned tomatoes, mashed potatoes, soups, and tomato sauces) constitutes 57% in France, 56% in the Republic of Ireland and the United Kingdom, 61% in the Netherlands, and 97% in Spain [40].

Lutein is a non-provitamin A carotenoid that belongs to the family of xanthophylls or oxycarotenoids [13]. It is distributed in a wide variety of vegetables, such as kale, spinach, and winter squash, and fruits such as mango, papaya, peaches, plums, and oranges [41]. Commercially, lutein is extracted from the flowers of the tagetes (*Tagetes erecta* L.), which contains 0.1–0.2% of carotenoids, of which 80 are diesters of lutein [42,43]. β-Cryptoxanthin, a xanthophyll with pro-vitamin A activity and one of the lesser-known carotenoids, is usually present in pumpkins, peppers, carrots, oranges, peaches, tangerines, and in tropical fruits such as papaya [39,44,45,46]. Table 1 shows the content of the six most important carotenoids in the diet for different fruits and vegetables [1,47,48].

The composition and content of carotenoids in fruits and vegetables is very variable, and depends on factors such as variety, genotype, season, geographical location/climatic conditions, soil, maturity stage, type of processing, and storage conditions [1,49,50]. Generally, carotenoid content in foods is not altered by common methods of cooking at home (microwave cooking, steaming, and boiling), but extreme heat can cause the oxidative destruction of carotenoids [51].

### 2.3. Bioavailability and Bioaccessibility

Before absorption, the carotenoids must be extracted from the food matrix in which they are ingested, transferred to the lipid emulsion, and incorporated into the micelles containing pancreatic lipases and bile salts [26,38,52]. Then, carotenoids are able to be transported to the enterocytes. However, their bioaccessibility in plants foods is remarkably low and these compounds are characterized by a slow absorption rate, since their chemical structure interacts deeply with the macromolecules within the food matrix of plants [53].

The factors that influence the bioavailability and bioaccessibility of carotenoids can be classified into two groups: (i) those related to carotenoids, which include dosage, chemical structure (isomeric forms), and interactions between carotenoids, and (ii) those not related to carotenoids, which include food processing and storage (raw, dehydrated, frozen, cooked), food composition, particle size of digested food, consumer biometrics, and transportation efficiency through the enterocyte [1,54,55,56,57,58,59]. Among these unrelated factors, thermal treatment increases the accessibility and bioavailability of carotenoids, due to the rupture of the cell walls and links with other macromolecules facilitating the release of carotenoids and improving their absorption [60,61,62]. The increase in the bioavailability of carotenoids when processing temperatures are above 100 °C (canning and sterilization) has been associated with isomerization, since the *cis* (*Z*) isomers are more bioavailable [1]. Several investigations have addressed the fact that the *Z*-isomerization of carotenoids influences not only bioavailability, but also antioxidant, anticancer, and antiatherosclerotic activities [63]. However, these results differ according to the type of carotenoid. For example, the *Z*-isomers of lycopene and astaxanthin have a greater bioavailability than the all-*E*-isomers [64,65], whereas the *Z*-isomers of β-carotene and lutein have a lower bioavailability than the all-*E*-isomers [66] It is important to understand the effect of *E*/*Z*-isomerization on functional changes, since this depends on its bioavailability and functionality of the carotenoids by ingestion [63]. In addition, mechanical processing, such as chopping and chewing, helps reduce the size of the particles and releases carotenoids from chloroplasts and tissues, increasing their bioavailability [67,68,69].

Another factor that influences the bioavailability of these compounds is the presence of other components of the diet. Thus, the presence of fat has a positive effect, and an intake of 3 to 5 g of fat is essential for the optimal absorption of carotenoids since it favors their incorporation into the micelle, facilitating their subsequent absorption [70,71]. Previous studies have even shown that long-chain fatty acids, such as oleic acid, are more beneficial for the absorption of non-polar carotenoids (carotenes) than non-polar ones (xanthophylls), by favoring their incorporation into the micelle [72,73]. On the other hand, the presence of dietary fiber and protein binding negatively affects their accessibility. Dietary fiber decreases the absorption of carotenoids by trapping them and interacting with bile acids, which leads to an increase in faecal excretion of fat and fat-soluble substances, such as carotenoids [57,74]. Protein–carotenoid complexes (such as lutein and zeaxanthin in spinach) and the microcrystalline form of some carotenoids (such as lycopene in tomatoes or β-carotene in carrots) makes them less available compared to those that are completely submerged in lipid droplets [75,76].

To assess bioavailability, the physiological state of the consumer must also be evaluated. The bioavailability of carotenoids can be modified in parasitic infestations (by intestinal helminths) and when there are diseases that produce intestinal dysfunction, as alterations in the uptake of carotenoids and in bioconversion have been observed [1]. In addition, age appears to be another factor contributing to the bioavailability of carotenoids, with a direct relationship between plasma carotenoid concentrations and the consumption of plant foods in groups of young adults, but not in advanced age groups [77], which suggests a lower bioavailability associated with age.

### 2.4. Nutritional Requirements 

To maintain a high content of carotenoids in the diet, dietary sources, the factors that influence their bioavailability, and the frequency of intake must be considered. As discussed above, serum carotenoid concentrations are used as a biomarker to establish the dietary intake of fruits and vegetables [78,79,80]. However, dietary intake and serum carotenoid levels show high variability between subjects from different populations, as well as between individuals from the same population [81,82], which may be due to the geographical availability of fruits and vegetables, socioeconomic status, and cultural factors [83]. Thus, in European countries, the total intake of carotenoids varies from 9.5 to 16 mg/day (3 to 6 mg/day for β-carotene), vegetables and fruits being the main dietary sources [84]. In the United States, the average intake of lycopene varies from 6.6 to 10.5 mg/day for men and 5.7–10.4 mg/day for women, of which more than 85% of the intake comes from tomatoes and tomato products (salsa, pasta, soup, juice, and ketchup) [85]. The intake of carotenoids in diets in various countries is shown in Table 2 [40,84,86].

Some in vivo studies have shown that exposure to high doses of carotenoids has a pro-oxidant effect. The beta-carotene and retinol efficacy trial (CARET) showed that participants who received a combination of β-carotene (30 mg) and vitamin A (25,000 IU retinyl palmitate) had incidences of lung cancer and mortality. These results are like those found for β-carotene in the alpha-tocopherol, beta-carotene (ATBC) study performed on 29,133 male smokers in Finland [87,88]. Haider et al. [89] also observed that high concentrations of β-carotene (50 µM) in primary pneumocyte type II cells produced a cytotoxic effect.

No specific recommendations regarding the intake of carotenoids have been published, apart from a daily recommendation for provitamin A carotenoids in the case of not consuming other sources of this vitamin. Thus, healthy adults are advised to consume 10.8–21.6 mg/day in order to reach the recommended daily dose of retinol (900 to 700 μg of equivalents/day) [90]. For the other carotenoids, some recommendations have been published according to their beneficial effect on health. Grune et al. [91] proposed a daily consumption of 7 mg of β-carotene to cover the basic need for this carotenoid. For lycopene, an intake of 5 to 7 mg per day was recommended for healthy people to maintain the circulating levels of this carotenoid, in order to combat oxidative stress and prevent chronic diseases [92]. Heath et al. [93] reported that higher concentrations of lycopene (35–75 mg/day) may be required when there is a disease, such as cancer and cardiovascular diseases. A daily intake level of up to 10 mg of lutein and zeaxanthin was recommended for the treatment of age-related early macular degeneration [94]. An intake of 3 mg/day of β-cryptoxanthin was suggested for the treatment of patients with NAFLD [17]. However, these recommendations are based on intervention studies and were proposed by researchers. Official dietary recommendations have not yet been issued by public health organizations.

## 3. Carotenoids and Hepatic Health

### 3.1. Pathogenesis of Non-Alcoholic Fatty Liver Disease (NAFLD)

The liver is the largest viscera in the body (weighing about 1.5 kg in a healthy adult) and is involved in numerous metabolic processes, such as the regulation of carbohydrates, lipids, and proteins. It also performs specific functions, such as synthesis of steroid hormones, detoxification of drugs, and conjugation of bilirubin [95].

The most common diseases of the liver are due to viral infections, alcohol consumption, autoimmune diseases, ischemia, and genetic disorders [96]. Obesity is also associated with liver damage and with an increased risk of NAFLD [97]. This disease affects 25%–45% of the general population and has a higher prevalence in diabetic and obese patients. Recent research has shown that in the United States, more than a third of adults and 17% of young people are obese. Among these, 70%–80% have NAFLD [98]. The prevalence of NAFLD in South America (evaluated by ultrasound) was estimated at around 30.45% and seems to be higher than the rate reported for the United States (20.0–29.9%). A meta-analysis published in 2016 reported an average prevalence of 23.71% in Europe, varying from 5–44% in different countries [99]. It is estimated that in the next 20 years, NAFLD will become the main cause of morbidity and mortality related to the liver and will be one of the main causes for liver transplantation [100].

NAFLD refers to the accumulation of excess fat in more than 5% of hepatocytes, without significant alcohol intake [101], and ranges from steatosis, with inflammation, to the progression to non-alcoholic steatohepatitis (NASH), fibrosis, cirrhosis, and in some cases, hepatocellular carcinoma [102]. The underlying mechanism for the progression of steatosis to inflammation and fibrosis is not fully understood, although insulin resistance, lipid metabolism disorders and oxidative stress are implicated [103,104,105]. Currently, the hypothesis that was proposed to explain the pathogenesis of NASH defends the existence of two impacts or hits; the first hit is due to insulin resistance and lipid overload, which leads to simple hepatic steatosis, and the second hit involves oxidative stress, lipid peroxidation, the induction of proinflammatory cytokines, and the inflammation process, which are the main causes that lead to the presence of NASH [104,106,107]. Lipid overload is caused by an increased entry of free fatty acids (FFA), leading to de novo lipogenesis. Insulin resistance, associated with the metabolic syndrome, also increases the accumulation of liver fat by increasing the release of free fatty acids and simulating anabolic processes [104,108]. The excess of FFA is stored in droplets within the hepatocyte, resulting in steatosis, which induces the innate immune response, with the recruitment of immune cells such as macrophages and T cells. As a result of the excess of intracellular fat and the deterioration of mitochondrial oxidative capacity, oxidation occurs in peroxisomes and microsomes, causing an increase in lipid peroxidation that leads to the generation of reactive oxygen species (ROS), damaging the proteins and the DNA. Kupffer cells (liver macrophages) produce proinflammatory cytokines, such as tumor necrosis factor α (TNF-α), in response to oxidative stress, mediating the inflammatory response that can cause cell death and damage. Thus, oxidative stress, together with inflammation, leads to fibrogenesis, a fundamental feature of the progression of steatosis to NASH [109].

**Figure 1:**
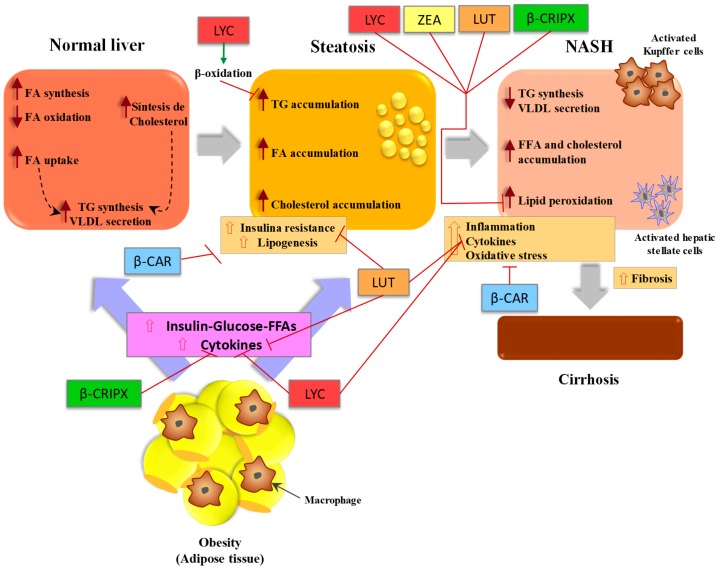
Diagram of the pathogenesis of non-alcoholic fatty liver disease (NAFLD) and the protective effect of carotenoids affecting different pathways. The red arrows denote blocked or decreased pathways, whereas the green arrows represent increased or promoted pathways. FA: fatty acids, TG: triglycerides, FFA: free fatty acids β-CAR: β-carotene, LYC: lycopene, LUT: lutein, β-CRIPX: β-cryptoxanthin, ZEA: zeaxanthin [19,110].

The mechanisms that represent the pathogenesis of NAFLD are presented in Figure 1 [19,110]. Although the pathogenesis and evolution of NAFLD to NASH is known, there is no agreement on the most effective pharmacological agents for its treatment. However, antioxidants such as carotenoids can play an important role in the defense against oxidative stress by avoiding or delaying oxidation, by neutralizing free radicals by sequestering singlet oxygen and inhibiting the progression of steatosis to steatohepatitis [2]. In fact, several studies have mentioned that carotenoids such as β-carotene, lycopene, lutein, and β-cryptoxanthin have antioxidant effects against lipid peroxidation in the liver of rats [111,112,113]. 

In addition, carotenoids in the diet, apart from being an important part of the antioxidant defense system, are also precursors of vitamin A, which can help rejuvenate the shape of hepatic stellate cells, preventing the progression of fibrosis to hepatocellular carcinoma [114]. A retrospective longitudinal study with 3336 middle-aged Chinese adults observed that the highest levels of serum carotenoids are associated with the improvement of the indicators of NAFLD, mediated by a reduction of the retinol binding protein 4 (*RBP4*), triglycerides, HOMA-IR, and body mass index [115]. The positive effects of carotenoids in the diet of preventing or treating NAFLD are their possible effects on hepatic health, individually, are taken into consideration and described below.

### 3.2. β-Carotene

This carotenoid has an important role as a precursor of vitamin A and has a direct impact on fighting against ROS, and hence protecting the body against oxidative stress [116,117]. Recent research has shown the possible preventive and protective effects of β-carotene on hepatic steatosis, fibrosis, oxidative stress, inflammation, and apoptosis [14]. In addition, this powerful antioxidant serves as a pre-hormone, since through metabolism, it is converted into retinoic acid, which functions as a ligand, regulating the expression of genes involved in metabolic processes [118].

Experimental studies have shown the potent hepatoprotective effect of β-carotene carried out in animal models, cell lines, and humans. Baybutt and Molteni [119] found that dietary supplementation of β-carotene had a protective effect on liver damage, demonstrating that rats with monocrotaline-induced steatosis decreased fat accumulation and liver hemorrhages. Patel and Sail [120] indicated that β-carotene protects physiological antioxidants against carcinogenesis induced by aflatoxin-B1 in albino rats. Another study with rats showed that supplementation with β-carotene increases the levels of vitamin C, glutathione, and enzymes related to glutathione, acting as scavengers of free radicals and consequently reducing the toxicity of aflatoxin-B1 [121]. In another animal study based on supplementation with (9*Z*)-β-carotene (isomer of β-carotene), a decrease of plasma cholesterol and atherogenesis index and a reduction of fat accumulation and inflammation was reported in the liver of mice fed a diet high in fat. This could be due to the transcriptional regulation of inflammatory cytokines, such as the vascular cell adhesion molecule 1 (*VCAM-1*), interleukin 1α (*IL-1α*), monocyte chemoattractant protein-1 (*MCP-1*), and interferon-γ (*INF- γ*) [122]. 

A study conducted by Ozturk et al. [123] found that the dietary intake of apricot reduced the risks of hepatic steatosis and the damage induced by carbon tetrachloride (CCl_4_) in Wistar rats. Markers of oxidative stress, such as malondialdehyde (MDA), total levels of glutathione (GSH), catalase, superoxide dismutase (SOD), and GSH peroxidase activities (GSH-Px), were significantly altered by CCl_4_. However, the liver damage and steatosis imposed by the high concentration of ROS were improved with the intake of apricots rich in β-carotene. Liu et al. [124] found that in a cell culture system, β-carotene could decrease the hepatosteatosis induced by the hepatitis C virus (HCV) by inhibiting RNA replication. Through its activity of provitamin A and its role in the inhibition of reactive oxygen species, β-carotene has been confirmed to have a positive effect on the progression of the hepatitis virus (HBV and HCV), preventing the development of carcinoma hepatocellular [125]. Another investigation showed that the Campari tomatoes, which contain more β-carotene and lycopene than normal tomatoes, improved diet-induced obesity, dyslipidemia, and hepatosteatosis through gene regulation related to lipogenesis in the model of zebrafish, transcriptionally lowering the expression of sterol regulatory element-binding transcription factor 1 (*SREBF1*) and increasing the expression of the forkhead box O1 gene (*FOXO1*) [126]. Other foods rich in β-carotene, such as goji berries (*Lycium barbarum*), have also improved liver fibrosis, oxidative stress, and inflammatory response in a rat model with NASH and cellular steatosis induced by a high-fat diet. These improvements were partially due to the modulation of the transcription factor NF-κB, the MAPK pathway, and the autophagic process [127]. 

A human investigation found that NAFLD has an inverse relationship with the nutritional status of vitamin A in individuals with class III obesity, observing low levels of retinol and serum β-carotene in patients with NAFLD, which entails a significant association between insulin resistance and retinol and β-carotene levels [128]. Moreover, a case-control study explored associations between dietary intake of vitamin A and carotenes (β-carotene), and the risk of primary liver cancer. They used a food frequency questionnaire to assess the usual dietary intake and through a logistic regression analysis, the researchers suggested that a higher dietary intake of retinol, carotene, and vitamin A (1000 μg RE/day) obtained from dietary sources was associated with a lower risk of primary liver cancer. The researchers also found that an intake of 2300 μg of RE/day of total vitamin A in the diet was the one with the lowest risk of primary liver cancer [129]. In addition, a recent study involving 62 patients with NAFLD and 24 control subjects showed that the serum levels of β-carotene and the ratio of β-carotene to retinol (SC/SR) in patients with NAFLD (hepatic steatosis, inflammation, and fibrosis) were significantly lower than in the controls. According to this, the researchers indicated that both β-carotene and SC/SR decreased gradually with the progression of the disease—from the normal liver, hepatic steatosis, to the limit of steatohepatitis. These results showed that a lower concentration of circulating β-carotene and an SC/SR ratio are associated with the histological severity of NAFLD [130].

### 3.3. Lycopene

The main protective effect of lycopene is due to its antioxidant effect through the inactivation of ROS and the extinction of free radicals [131]. Beyond its antioxidant capacity, there are many other potential non-antioxidant mechanisms, of which lycopene can protect against chronic diseases, including the regulation of gene expression, gap junctions, antiproliferative capacity, immune and hormonal modulation, among others [23,132,133]. This is why lycopene is one of the most studied carotenoids in the prevention and treatment of NAFLD [14].

It has been confirmed that this antioxidant has a potential hepatoprotective effect in hepatitis induced by D-galactosamine/lipopolysaccharide (D-GaIN/LPS) in rats, affecting the metabolism of lipoproteins, restoring the altered levels of lipid metabolizing enzymes, and stabilizing the disposition of lipoprotein levels [134,135]. A study conducted by Wang et al. [136] investigated the protective effect of the intake of lycopene and tomato extract in the hepatocarcinogenesis promoted by NASH in an in vivo study. In this study, Sprague-Dawley rats were used, and a single dose administration of diethylnitrosamine (DEN) was applied because of its origin in a hepatocellular carcinoma. Note that lycopene and tomato extract can inhibit hepatocarcinogenesis in relationships through the reduction of oxidative stress. In addition, a significant decrease in cytochrome P450 2E1, inflammatory foci, and mRNA expression of proinflammatory cytokines (*TNF-α*, *IL-1β*, and *IL-12*) were also found. A study conducted by Ahn et al. [137] indicated that lycopene altered the down regulation of the expression of miRNA-21 (*miR-21*) in mice, induced by a high-fat diet. As a regulator of gene expression at the posttranscriptional level, miR-21 was upregulated by the ingestion of lycopene, inhibiting the expression of the fatty acid-binding protein 7 (*FABP7*) and blocking the accumulation of intracellular lipids induced by stearic acid in Hepa 1–6 cells. It was indicated that lycopene prevented non-alcoholic steatohepatitis in rats and mice, which was induced by a high-fat diet, and a reduction in oxidative stress in cells was observed [138,139,140]. This demonstrates that the incorporation of this carotenoid in a balanced diet prevents NAFLD [16,141]. Kujawska et al. [142] noted that tomato paste intake in rats before administration of N-nitrosodiethylamine (NDEA) was effective in recovering the enzymes SOD, catalase, and glutathione reductase by 32%–97%, indicating the protective role against oxidative stress induced by NDEA. In addition, the study showed that DNA damage induced by NDEA in leukocytes decreased by 10% in rats treated with tomato paste. It has been suggested that lycopene supplementation in the diet prevents the incidence of hepatocellular carcinoma (HCC) induced by high-fat diets in mice, suppressing oncogenic signals, including methionine mRNA, β-catenin protein, and the activation of complex 1 of the target of rapamycin in mammalian cells (mTOR). This suggests that lycopene in the diet and its metabolites can be used in the prevention of liver cancer in patients with NAFLD [143,144]. Martín-Pozuelo et al. [145] studied the effect of tomato juice intake on gene expression in rats with induced hepatic steatosis, noting that supplementation with tomato juice led to an accumulation of all-*E* and *Z*-lycopene, as well as their metabolites in the liver of animals fed a normal diet + lycopene (NL) and a high-fat diet + lycopene (HL), with higher levels in the treatment of HL than in the NL group (63.07% vs. 44.45%) due to a higher absorption. In addition, it was shown that rats fed high-fat diets and tomato juice compared to rats that ingested only high-fat diets and water (NA) had significantly increased levels of high-density lipoproteins (HDL), and this also decreased oxidative stress through the reduction of isoprostanes in the urine. Regarding the analysis of gene expression of biomarkers associated with lipid metabolism, the overexpression of several genes related to the transport of fatty acids, lipid hydrolysis, and β-oxidation of mitochondrial and peroxisomal fatty acids was observed. In vitro and in vivo studies demonstrated that lycopene reduces ROS production in SK-Hep-1 cells by inhibiting dicotinamide adenine dinucleotide phosphate oxidase (NADPH) through protein kinase C (PKC) signaling. Furthermore, it was indicated that lycopene improved hepatotoxicity by acting as an antioxidant, mainly by reducing protein carbonylation and areas of necrosis, ameliorating the general appearance of the lesion in C57BL/6 mice [146]. A recent study showed that lycopene exerted anti-inflammatory activities against paracetamol liver injury (APAP) in C57BL/6 mice by improving the redox state [147]. Yefsah-Idres et al. [148] showed that rats with a diet high in methionine content had abnormal histological features accompanied by an increase in the levels of serum homocysteine, alanine aminotransferase (ALT), and aspartate aminotransferase (AST), as well as MDA hepatic and a decrease in the activities of cystathionine-β-synthase (CBS) and S-adenosyl-homocysteine hydrolase, indicating that lycopene supplementation reversed hyperhomocysteinemia (related to oxidative stress), providing additional evidence of the hepatoprotective effects of lycopene. Lycopene also showed beneficial effects against HCC by modulating cell proliferation, glycolysis, and ultrastructure of liver cells [149]. Xu et al. [150] confirmed that lycopene alleviates liver injury induced by aflatoxin B1 (AFB1) by improving hepatic oxidation and detoxification potential with Nrf2 activation. In another model of NAFLD and hypercholesterolemia induced by a high-fat diet, researchers showed that consumption of tomato juice had different effects depending on the diet. In the group of rats that were taking tomato juice (with and without steatosis), the genes involved in β-oxidation as well as the thrombospondin receptor (*CD36*) were positively regulated, and apolipoprotein B (*APOB*) and lipoprotein lipase (*LPL*) were negatively regulated. The accumulation of lycopene in rats with steatosis positively regulated the farnesoid X-activated receptor (*FXR*) and the hepatocyte nuclear factor 4 alpha (*HNF4A*), which have been suggested as preventive factors in relation to steatosis [151]. Regarding the metabolomic study, the intake of tomato juice in rats with fat-induced steatosis stimulated the biosynthesis of glutathione and the amino acids of the transulfurization pathway, increasing the levels of metabolites related to the antioxidant response [139,151].

### 3.4. Lutein

The property of this antioxidant is also based on the uptake of free radicals, especially singlet oxygen, protecting against oxidative damage [152], although an antiviral activity against hepatitis B has also been described, since it inhibits the transcription of the virus [153]. Kim et al. [154] observed that lutein (0.1 g/100 g for 12 weeks) decreased inflammation and oxidative stress in the liver and in the eyes of guinea pigs fed a hypercholesterolemic diet. This carotenoid could prevent the degenerative conditions of the liver by decreasing the accumulation of free cholesterol, attenuate lipid peroxidation (decreased MDA), and the production of proinflammatory cytokines (TNF-α). Furthermore, in this study, it was also observed that guinea pigs fed lutein also had a lower DNA-binding activity of NF-κB. These antioxidant effects suggest protective effects against NAFLD. A protective anticarcinogenic effect after NDEA induction in rats with HCC was described, noting that the administration of lutein inhibited carcinogenesis, probably due to the combination of its antioxidant activity and the activation of cytochrome P450 enzymes, as well as other detoxifying enzymes such as glutathione S-transferase (GST) and UDP-glucuronyl transferase [155]. Another study suggested that lutein supplementation may protect against hepatic lipid accumulation and insulin resistance induced by a high-fat diet. In addition, the study also investigated the effects of lutein on the expression of the peroxisome proliferator activated receptor (PPAR) because it plays an important role in lipid metabolism, finding that the high-fat diet significantly inhibited the expression of the PPAR, which was restored with lutein supplementation [156]. Murillo et al. [157] used a nanoemulsion of lutein (3.5 mg/day) in a hypercholesterolemic diet administered to guinea pigs during six weeks, and observed an increase in the concentrations of this carotenoid in plasma and liver, in addition to a point decrease in hepatic steatosis (24% lower as assessed histologically), total liver cholesterol, and plasma ALT activity. In addition, in the study, a 55% decrease in LDL was also found in the groups supplemented with lutein compared to the control groups. These results suggest the protective effects of this nanoemulsion on hepatic steatosis.

### 3.5. β-Cryptoxanthin

According to its antioxidant activity, plasma concentrations of β-cryptoxanthin are inversely related to oxidative DNA damage rates and lipid peroxidation [158]. In addition, in in vivo and in vitro studies, it was observed that β-cryptoxanthin has anti-inflammatory effects, modulating the immune response of macrophages [159]. Takayanagi et al. [160] demonstrated that oral administration of this carotenoid repressed the secretion of proinflammatory cytokines (TNF-α, IL-1, and IL-6) and improved lipid metabolism and energy consumption. Kobori et al. [161] demonstrated that β-cryptoxanthin improved dietary-induced NASH by suppressing the expression of inflammatory genes in mice. They observed that this carotenoid suppressed the expression of genes inducible by LPS and by TNF-α in NASH. The elevated levels of the thiobarbituric acid reactive substances (TBARS) of the oxidative stress marker were also reduced. Therefore, β-cryptoxanthin represses inflammation and the resulting fibrosis, probably by suppressing the increase and activation of macrophages/Kupffer cells, leukocytes, and T cells. In a model with mice (lipotoxic model), it was observed that β-cryptoxanthin reversed steatosis, inflammation, and progression of fibrosis in NASH, reversing insulin resistance and preventing steatohepatitis by decreasing the activation of macrophages or Kupffer cells [162]. Another study showed that supplementation with β-cryptoxanthin in patients with NAFLD inhibited the progression of this disease, suggesting that the intake of β-cryptoxanthin is very effective in elevating antioxidant and anti-inflammatory activities in patients with NAFLD (17).

### 3.6. Other Carotenoids

Other carotenoids, such as α-carotene and zeaxanthin, also showed beneficial effects against chronic liver injury. An investigation carried out by Murakoshi et al. [163] found that α-carotene had an inhibitory effect on spontaneous hepatic carcinogenesis in male mice, significantly decreasing the mean number of hepatomas. Zeaxanthin showed protective effects against NAFLD, decreasing oxidative stress and liver fibrosis, suggesting that the mechanism of action of zeaxanthin is related to its antioxidant capacity [164]. Epidemiological studies showed that zeaxanthin is inversely associated with the prevalence of NAFLD in Chinese populations of medium and advanced ages [165]. A summary of the studies examining the role of these carotenoids in chronic liver diseases is described in Table 3.

## 4. Conclusions

The available evidence regarding the potential use of dietary carotenoids in liver health suggests that these compounds are effective in reducing lipid accumulation, insulin resistance, oxidative stress, and inflammation of hepatocytes, which is why they could be used as a dietary alternative for the prevention and treatment of NAFLD. The effects of the specific mechanisms by which carotenoids protect against NAFLD are depicted in Figure 1. The antioxidant and anti-inflammatory properties are the main mechanisms of action of carotenoids, modulating intracellular signaling pathways that influence gene expression and protein translation. During the last decade, several investigations were carried out, attempting to elucidate the protective function of carotenoids against the lesions induced by oxidative stress, especially the anticancer effects, by preventing tumors and modulating the proliferation of liver cells. In addition, several carotenoids have provitamin A activity, which helps to rejuvenate the shape of hepatic stellate cells and prevent the progression of fibrosis to HCC. More preclinical and clinical studies are needed to evaluate if there could be an effective dose, considering the bioavailability of dietary carotenoids, for the prevention of NAFLD.

## Figures and Tables

**Table 1:** Data on the contents of major carotenoids in fruits and vegetables common in the human diet (mg/100 g) [1,47,48].

Food	Lutein	Zeaxanthin	β-Cryptoxanthin	α-Carotene	β-Carotene	Lycopene
Avocado	0.21–0.36	0.01	0.02–0.03	0.02–0.03	0.05–0.08	–
Banana	0.09–0.19	–	n.d.–0.01	0.06–0.16	0.04–0.13	n.d.–0.25
Peach	–	–	–	–	–	0.01
Guava	–	–	0.02–0.12	n.d.	0.10–2.67	0.77–1.82
Fig	0.08	–	0.01	0.02	0.04	0.32
Kiwi	–	–	–	–	<0.02	<0.01
Mandarin Orange	–	–	0.63–1.06	n.d.	0.11–0.32	–
Mango	–	–	0.02–0.32	n.d.	0.11–1.20	<0.01–0.72
Apple	0.02	n.d.	n.d.	n.d.	0.019	n.d.
Passion fruit	–	–	0.18	–	0.36–0.78	–
Orange	–	–	0.07–0.14	n.d.	0.17–0.48	n.d.
Peach	–	0.02–0.04	0.004–0.02	–	0.14–0.26	–
Papaya	0.09–0.32	–	n.d.–1.03	n.d.	0–08–0.66	n.d.–7.56
Pineapple	–	–	0.07–0.12	n.d.	0.14–0.35	0.27–0.61
Watermelon	–	–	n.d.	n.d.	0.31–0.78	4.77–13.52
Grapefruit	–	–	–	–	–	0.75
Tangerine	0.17	il	0.43	0.03	0.26	–
Grape	0.01	n.d.	n.d.	n.d.	0.02	n.d.
Plum	0.08–0.09	n.d.	n.d.	n.d.	0.09–0.14	n.d.
Apricot	0.12–0.19	n.d.–0.04	–	n.d.–0.04	0.59–3.80	0.05
Chard	3.60	0.01	n.d.	n.d.	2.90	n.d.
Artichoke	0.59–0.63	–	–	–	0.27–0.37	–
Broccoli	0.71–3.30	–	n.d.	n.d.	0.29–1.75	n.d.
Pumpkin	0.63	–	0.06	–	0.49	0.50
Sweet Potato	0.05	–	–	–	7.83	–
Peas	1.91	il	n.d.	n.d.	0.52	n.d.
Red Pepper	0.25–8.51	0.59–1.35	0.25–0.45	n.d.–0.29	1.44–2.39	–
Jalapeño Pepper	0.84	–	–	0.01–0.17	0.38–8.58	–
Spinach	5.93–7.90	il	n.d.	n.d.	3.10–4.81	n.d.
Lettuce	1.00–4.78	–	–	–	0.87–2.96	–
Corn	0.41	0.22	n.d.	n.d.	n.d.	n.d.
Cucumber	0.46–0.84	il	n.d.	n.d.	0.11–0.27	n.d.
Red chili	n.d.	–	–	–	6.53–15.40	–
Cabbage	0.45	il	n.d.	n.d.	0.41	n.d.
Tomato	0.05–0.21	il	n.d.	n.d.	0.32–1.50	0.85–12.70
Carrot	0.25–0.51	il	n.d.	2.84–4.96	4.35–8.84	n.d.
Kale	4.80–11.47	–	–	–	1.02–7.38	–
Parsley	6.40–10.65	il	n.d.	n.d.	4.44–4.68	n.d.
Coriander	6.00–14.80	–	–	2.90–11.30	4.80–8.40	–

–: not included in the references, n.d.: not detected or quantified, il: included in lutein.

**Table 2:** Dietary consumption of carotenoids in different countries (data are reported as mean and [median]) [40,84,86].

Sample (N), Country	Woman/Man (Age)	Dietary Intake (mg/day)					
		α-car	β-car	β-cryp	Lut/ Zea	Lyco	Total
EUROPE							
N = 1968, Italy	W, M (> 1)	0.15	2.6	0.17	4.01	7.38	14.31
N=75, France	W, M (25–45)	[0.74]	[5.84]	[0.45]	[2.50]	[4.75]	14.28
N = 65, North Ireland	W, M (25–45)	1.04	5.55	0.99	1.59	5.01	14.18
N = 71, United Kingdom	W, M (25–45)	[1.04]	[5.55]	[0.99]	[1.59]	[5.01]	14.18
N = 73, Ireland	W, M (25–45)	1.23	5.16	0.78	1.56	4.43	13.16
N = 72, Netherlands	W, M (25–45)	0.68	4.35	0.97	2.01	4.86	12.87
N = 159, Sweden	W (56–75)	1.03	3.47	0.46	2.64	2.15	9.75
N = 3000, Spain	W, M (18–64)	0.27	1.46	0.32	1.24	3.06	6.35
OCEANIA							
N = 91, Australia	W (18–70)	[2.0]	[6.87]		[2.28]	[5.05]	16.2
AMERICA							
N = 459, Costa Rica	115 W (59±10) 344 M (56±11)	0.73 0.45	4.67 3.41	0.55 0.38	2.89 2.41	5.77 5.45	14.61 12.10
N = 402, USA (Afro-American)	155 M (34–84) 247 W (34–84)	[0.33] [0.25]	[2.21] [2.21]	[0.11] [0.13]	[1.85) [1.93]	[3.16] [2.60]	7.66 7.12
N = 50, Dominican Republic	W, M (50–90)	0.7	2.7	0.22	1.33	1.46	6.41
USA	W, M (≥ 20)	0.4	1.9	0.2	1.4	1.4	5.3
N = 55,950, Brazil	W, M (≥ 10)	0.16	0.92	0.16	0.83	0.83	2.9

**Table 3:** Summary of studies in which carotenoids had a beneficial effect on chronic liver diseases in cell lines, and human and animal models.

Agent	Model	Main Results	Reference
β-carotene	Rat: carcinogenesis induced by AFB1	↑ Antioxidantes enzymes (GSH-Px, catalase, GST) and vitamin C ↓ Risk of toxicity due to AFB1	[120]
Alga *Dunaliella bardawil* (rich in (9*Z*)-β-carotene)	Mouse: fed high-fat diet, LDL receptor knockout mouse	↓ Plasma cholesterol and atherogenesis (VLDL y LDL) ↓ Accumulation of fat and liver inflammation ↓ Levels of hepatic inflammatory genes (*VCAM-1*, *IL-1α*, *MCP-1*, *INF-γ*)	[122]
Apricot (rich in β-carotene)	Rat: Hepatic steatosis and damage induced by CCL_4_	↓ Liver MDA ↑ Levels of total GSH, catalase, SOD and GSH-Px ↓ Oxidative stress ↓ Hepatic steatosis and liver damage	[124]
Tomato “Campari” (rich in β-carotene and lycopene)	Zebrafish: Obesity induced by diet	↓ *SREBF1* in the Marn ↑ *FOXO1* in the expression of genes ↓ Diet-induced obesity, dyslipidemia and hepatic steatosis	[127]
*Lycium barbarum* polysaccharides (rich in β-carotene)	Rat: NASH induced by a high-fat diet	↑ Modulation of NF-κB and the MAPK pathway ↓ Accumulation of liver fat, inflammatory liver response, fibrosis and oxidative stress ↑ Hepatoprotective properties	[128]
Dietary carotenes and vitamin A	Human: patients with primary liver cancer	↓ Risk of primary liver cancer	[129]
Lycopene	Rat: NASH induced by high-fat diet	↓ Levels of CYP2E1 protein, MDA (plasma and liver) and TNF-α ↑ Hepatic GSH level ↓ Steatosis and inflammation	[138]
Tomato juice	Rat: hypercholesterolemic and NAFLD induced by the diet	↓ Levels of TG in plasma and isoprostanes in urine ↑ Acummulation of lycopene in the liver ↑ Relief of amino acid depletion ↑ Recovery of the redox balance in the liver ↑ Levels of L-carnitine ↑ Protective effect of NAFLD	[139]
Tomato juice	Rat: NAFLD induced by a high-fat diet	↓ Isoprostanes in urine, plasma TG and LDL ↑ Activity of mitochondrial β-oxidation and peroxisomal ↓ Steatosis	[145]
Lycopene	SK-Hep-1 cells: PKC pathway mediated by ROS production. Mouse: Hepatotoxicity induced by APAP overdose	↓ Production of ROS, NADPH oxidase and *MMP-2*, GSSG ↑ GSH and CAT	[146]
Lycopene	Rat: NAFLD induced by a high-fat diet	↓ ALT, AST, triglyceride, total cholesterol, MDA, LDL and FFA ↓ *CYP2E1* and *TNF-α* ↑ GSH, SOD y HDL ↑ Protective effect on NAFLD	[16]
Lycopene	Rat: NAFLD induced by a high-fat diet	↓ liver weight, LDL and liver total cholesterol ↑ GSH-Px, SOD and CAT en the liver	[141]
Lycopene	Mouse: liver injury induced by AFB1	↓ Acummulatio of AFB1-ADN adducts in the liver ↑ Activation of Nrf2 signaling ↑ Antioxidant potential and liver detoxification	[150]
Tomato juice	Rat: hypercholesterolemic and NAFLD induced by the diet	↑ Regulation of *CD36*, *FXR* and *HNF4A* ↓ Regulation of *APOB* and *LPL* ↓ Synthesis of fatty acids, triglycerides and cholesterol ↑ Levels of metabolites related to the antioxidant response	[151]
Lutein	Guinea pig: Hepatic steatosis induced by a hypercholesterolemic diet	↓ Hepatic free cholesterol ↓ Malondialdehyde and hepatic TNF-α ↓ Binding to the hepatic DNA of NF-κB	[154]
Lutein	Rat: Hepatocellular carcinoma induced by *N*-nitrosodiethylamine (NDEA)	↓ ALT, AST, alkaline phosphatase in plasma and liver tissue ↑ GSH ↓ GGT ↑ UDP-glucoronyl transferase and glutathione-S-transferase	[155]
Lutein	Rat: NAFLD induced by a high-fat diet	↓ Liver total cholesterol and triglycerides ↑ HDL in serum ↓ ALT in serum ↑ Hepatic insulin sensitivity ↑ Hepatic fatty acids catabolism	[156]
Lutein	Guinea pig: Hepatic steatosis induced by a hypercholesterolemic diet	↓ Hepatic steatosis (evaluated histologically) ↓ Total hepatic cholesterol ↓ Plasma ALT and LDL activity	[157]
β-cryptoxanthin	Mouse: Obese model	↓ Body weight and abdominal adipose tissue ↓ Triglycerides and serum total cholesterol ↓ Inflammatory citokines ↑ Lipid metabolism and energy consumption	[160]
β-cryptoxanthin	Mouse: NASH induced by a diet high in cholesterol and high in fat	↓ Liver TBARS ↑ Suppresses the expression of the inducible *LPS* and *TNF-α* genes ↓ Inflammatory response (suppresses the activation of macrophages, T helper and citototoxic cells)	[161]
β-cryptoxanthin	Mouse: Hepatic steatosis and NASH induced by the diet high in fat and cholesterol	↓ Total content of hepatic macrophages and T cells	[162]
β-cryptoxanthin	Human: Patients with NAFLD (NASH and NAFL)	↓ GGT, LDL and serum IL-6 ↑ SOD and serum IL-10 ↑ Antioxidant and anti-inflammtory activities	[17]
α-carotene	Mouse: spontaneous hepatic carcinogenesis	↓ Hepatomas	[163]
Zeaxanthin	Gerbil from Mongolia: NASH induced by a diet deficient in methionine and choline	↓ Liver fibrosis ↓ Hepatic lipid hydroperoxides	[164]

## Abbreviations

MAPK	Mitogen-activated protein kinase
CYP2E1	Cytochrome P450 family 2 subfamily E member 1
LDL	Low density lipoproteins
HDL	High density lipoprotein
Nrf2	Nuclear factor erythroid 2-related factor 2
GGT	Gamma-glutamyltransferase
UDP	Uridine diphosphate
IL-6	Interleukin-6
IL-10	Interleukin-10
NF-κB	Nuclear factor kappa B
